# Mutant B2M‐HLA‐E and B2M‐HLA‐G fusion proteins protects universal chimeric antigen receptor‐modified T cells from allogeneic NK cell‐mediated lysis

**DOI:** 10.1002/eji.202049107

**Published:** 2021-08-19

**Authors:** Yelei Guo, Beilei Xu, Zhiqiang Wu, Jian Bo, Chuan Tong, Deyun Chen, Jin Wang, Haoyi Wang, Yao Wang, Weidong Han

**Affiliations:** ^1^ Department of Bio‐therapeutic the First Medical Centre Chinese PLA General Hospital Beijing China; ^2^ State Key Laboratory of Stem Cell and Reproductive Biology Institute of Zoology Chinese Academy of Sciences Beijing China; ^3^ Department of Hematology the First Medical Centre Chinese PLA General Hospital Beijing China; ^4^ Department of Outpatient the Sixth Medical Centre Chinese PLA General Hospital Beijing China

**Keywords:** chimeric antigen receptor, HLA‐E, HLA‐G, NK cells, universal CAR T

## Abstract

Recent studies have indicated the antitumor activity and reduced allogeneic response of universal chimeric antigen receptor‐modified T (UCAR T) cells lacking endogenous T cell receptors and beta‐2 microglobulin (B2M) generated using gene‐editing technologies. However, these cells are vulnerable to lysis by allogeneic natural killer (NK) cells due to their lack of human leukocyte antigen (HLA) class I molecule expression. Here, constitutive expression of mutant B2M‐HLA‐E (mBE) and B2M‐HLA‐G (mBG) fusion proteins in anti‐CD19 UCAR T (UCAR T‐19) cells was conducted to protect against allogeneic NK cell‐mediated lysis. The ability of cells expressing mBE or mBG to resist NK cell‐mediated lysis was observed in gene‐edited Jurkat CAR19 cells. UCAR T‐19 cells constitutively expressing the mBE and mBG fusion proteins were manufactured and showed effective and specific anti‐tumor activity. Constitutive expression of the mBE and mBG fusion proteins in UCAR T‐19 cells prevented allogeneic NK cell‐mediated lysis. In addition, these cells were not recognizable by allogeneic T cells. Additional experiments, including those in animal models and clinical trials, are required to evaluate the safety and efficacy of UCAR T‐19 cells that constitutively express mBE and mBG.

## Graphical abstract

UCAR T cells, which could not cause graft‐versus‐host‐disease (GvHD) and host‐versus‐graft (HvG) through knocking out endogenous TCR and MHC class I molecules, express mutant B2M‐HLA‐E (mBE) and B2M‐HLA‐G (mBG) fusion proteins protecting against allogeneic NK‐mediated lysis and enhancing their survival persistence and anti‐tumor ability in vivo (Image created with BioRender.com).

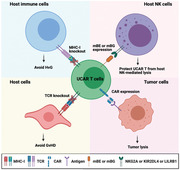

## Introduction

Recent immunotherapy using autologous anti‐CD19 chimeric antigen receptor‐modified T (CAR T‐19) cells has shown great success in leukemia [1, 2, 3]. Nevertheless, manufacturing a sufficient number of effective autologous CAR T cells for the treatment of cancer patients is not always possible for several reasons, including intensive treatment before recruitment and an insufficient number and poor quality of T cells [2, 4]. Allogeneic CAR T cells derived from healthy donor T cells may have clinical efficacy, but challenges remain [3, 5]. However, the process for manufacturing allogeneic CAR T cells is tailored, expensive, and time consuming, and graft‐versus‐host‐disease (GvHD) can be induced after allogeneic CAR T cell infusion [3, 6]. These limitations may restrict the clinical application of autologous and allogeneic CAR T cells.

Universal CAR T cells were recently indicated to be effective in treating cancer patients and engineered to lack endogenous T cell receptors (TCRs) using gene‐editing technologies to reduce the occurrence of GvHD [7, 8, 9]. However, allogeneic T cells can be rapidly rejected by the host's immune system because of their expression of human leukocyte antigen (HLA). To preclude mismatches between donor and recipient HLA class I molecules, disrupting beta‐2 microglobulin (B2M), an essential subunit of class I molecules, could sustain allograft survival [10, 11, 12]. Although CAR T cells lacking both TCRs and HLA class I molecules exhibit reduced alloreactivity and are less likely to cause GvHD, HLA class I‐negative cells can still be lyzed by natural killer (NK) cells of the host [10, 13, 14]. The minimally polymorphic HLA‐E and HLA‐G proteins, nonclassical HLA class I molecules, are ligands for the NK cell inhibitory receptor and may therefore prevent NK cell‐mediated lysis of HLA class I molecule‐negative cells without alloreactivity [15, 16]. Therefore, engineering TCR and HLA class I molecule‐double deficient CAR T cells to express HLA‐E or HLA‐G may be worthwhile to prohibit allogeneic NK cell‐mediated lysis.

Herein, we used lentiviruses and CRISPR/Cas9 gene‐editing system to develop TCR and HLA class I molecule double‐knockout (DKO) universal CAR T‐19 (UCAR T‐19) cells that expressed a mutant B2M‐HLA‐E (mBE) or mutant B2M‐HLA‐G (mBG) fusion protein. We showed that the expression of mBE and mBG fusion proteins in UCAR T‐19 cells prevented allogeneic NK cell‐mediated lysis and were not recognizable by allogeneic T cells, indicating their potential for use in cancer treatment applications.

## Results

### Efficient generation of TCR and B2M double‐gene knockout CAR‐positive Jurkat cells

**Figure 1:**
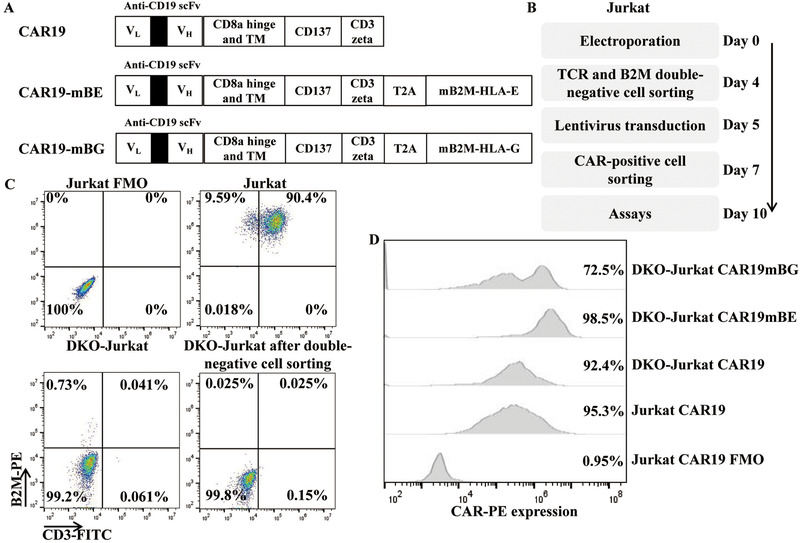
Generation of gene‐edited Jurkat CAR19 cells. (A) Schematic representation of a panel of chimeric receptors containing anti‐CD19 CAR and different fusion proteins. The fusion proteins contained mutant B2M and HLA‐E or mutant B2M and HLA‐G. (B) TCR and B2M DKO Jurkat CAR19 cells were produced and cultured for 10 days before being used in subsequent assays. (C) TCR and B2M expression in Jurkat and gene‐edited Jurkat cells before and after TCR and B2M double‐negative cell sorting. Gates were established by using fluorescence‐minus‐one (FMO) controls (CD3 and B2M). The numbers represent the percentages of positive cells. (D) Representative CAR expression in gene‐edited Jurkat CAR19 cells. Gates were established by using FMO controls (biotin‐SP‐AffiniPure F(ab)’2 fragment).

In an attempt to develop UCAR T‐19 cell therapies, we designed a second‐generation, CD19‐specific CAR lentiviral vector harboring the single‐chain fragment variable (scFv) derived from GenBank no. HM852952.1 [3] as well as the CD137 and CD3zeta signaling domains. In addition, the CAR19‐mBE or CAR19‐mBG lentiviral vector encoded a CAR19 paralleling an mBE or mBG fusion protein was engineered using a self‐cleaving T2A peptide configuration. A schematic representation of the lentiviral vector constructs used in this study is shown in Fig. 1A.

The detailed procedure for manufacturing gene‐edited Jurkat CAR19 cells is presented in Fig. 1B. First, Jurkat cells were electroporated with TRAC gRNA, B2M gRNA, and the Cas9 protein and then transduced with lentivirus after TCR and B2M double‐negative cell sorting (Fig. 1C and Supporting Information Fig. 1A). As shown in Fig. 1D, high levels of CAR expression were observed after culturing for 10 days.

### Expression of mBE and mBG in gene‐edited Jurkat cells protected against NK cell‐mediated lysis

**Figure 2:**
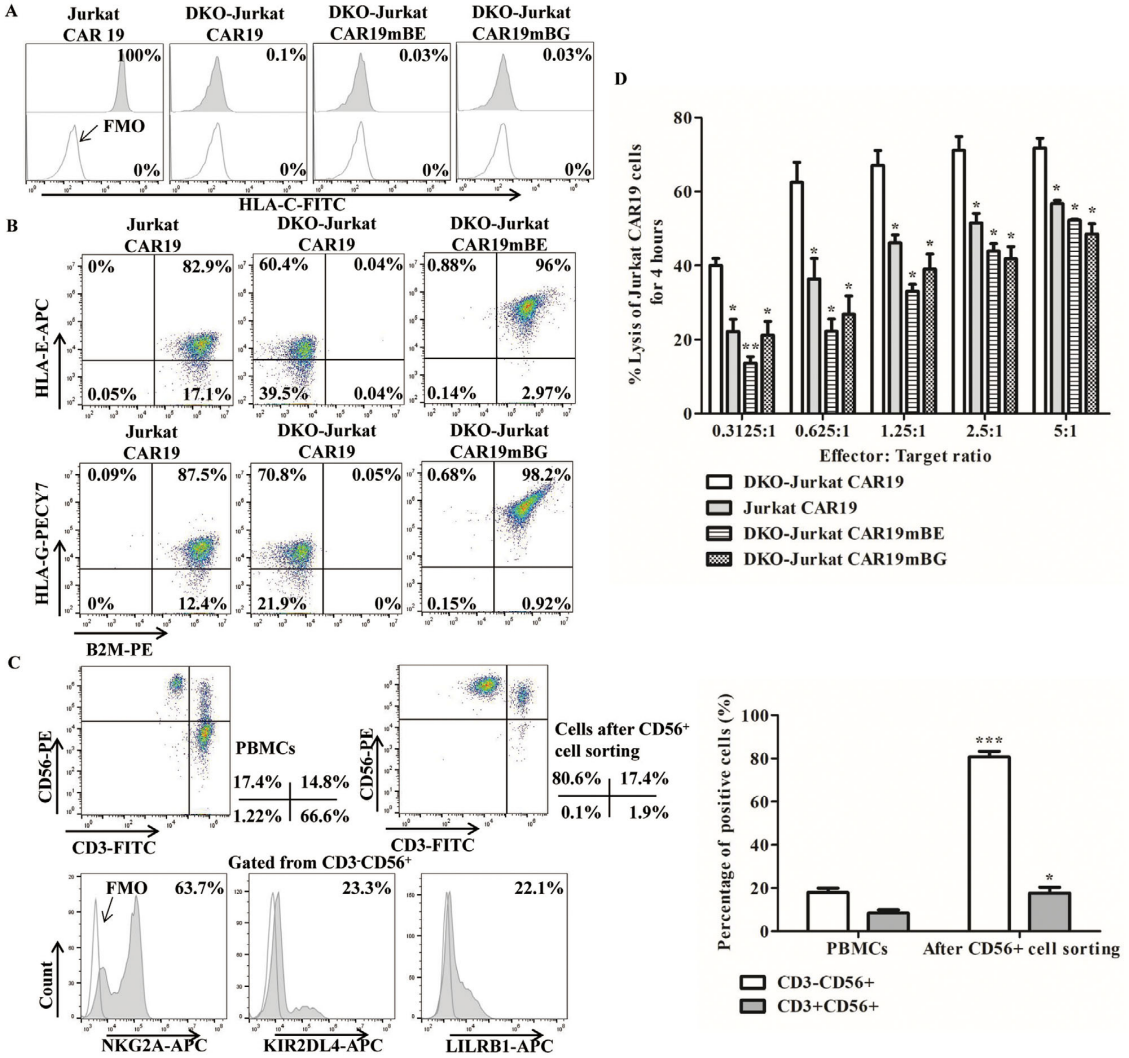
Constitutive expression of the mBE and mBG fusion proteins in DKO gene‐edited Jurkat CAR19 cells provide protection against allogeneic NK cell‐mediated lysis. (A) Representative HLA‐C expression in DKO gene‐edited Jurkat CAR19 cells. Gates were established by using FMO controls (HLA‐C). (B) Representative expression of HLA‐E and HLA‐G in DKO gene‐edited Jurkat CAR19 cells. (C) CD56^+^ NK cells were isolated from PBMCs harvested from six healthy volunteer donors. The panels on the left upper show the CD3 and CD56 expression in PBMCs and CD3^−^CD56^+^ cells after CD56^+^ cell sorting. The statistical analysis of CD3^−^CD56^+^ cells after CD56^+^ cell sorting is shown on the right panel (*n* = 6 different donors in three independent experiments. *P < 0.05, ***P < 0.001, versus PBMCs; *t*‐test, GraphPad Prism version 5). The panels on the left lower show the expression of the killer‐cell immunoglobulin‐like receptors NKG2A, KIR2DL4, and LILRB1 in CD3^−^CD56^+^ NK cells; gates were established by using FMO controls (NKG2A, KIR2DL4, and LILRB1). The numbers represent the percentages of positive cells. (D) NK cell cytotoxicity in DKO gene‐edited Jurkat CAR19 cells after cocultured at effector‐to‐target ratios of 5:1, 2.5:1, 1.25:1, 0.625:1, and 0.3125:1 for 4 h (*n* = 3 different donors in three independent experiments. *P < 0.05, **P < 0.01, versus DKO‐Jurkat CAR19; *t*‐test, GraphPad Prism version 5).

To further investigate the ability of cells expressing mBE or mBG to resist NK cell‐mediated lysis, we first evaluated the HLA expression in TCR and B2M DKO gene‐edited Jurkat CAR19 cells. Our editing strategy produced DKO Jurkat CAR19 cells that were HLA‐C negative, unlike Jurkat CAR19 cells (Fig. 2A). Then, the HLA‐E and HLA‐G expression in DKO Jurkat CAR19 cells was evaluated (Fig. 2B). The DKO‐Jurkat CAR19mBE and DKO‐Jurkat CAR19mBG cells had a higher mean fluorescence intensity (MFI) and percentages of HLA‐E and HLA‐G than Jurkat CAR19 and DKO‐Jurkat CAR19 cells.

Next, we determined the expression of the mBE and mBG fusion proteins in gene‐edited Jurkat CAR19 cells to evaluate their ability to prevent allogeneic NK cell‐mediated lysis. HLA‐E binds to the inhibitory receptor NKG2A, and HLA‐G binds to the inhibitory receptors KIR2DL4 and LILRB1, both of which are expressed in most human NK cells [17, 18, 19, 20]. Allogeneic peripheral blood primary CD56^+^ NK cells isolated from healthy volunteer donors were cocultured with Jurkat CAR19 and DKO‐Jurkat CAR19 cells (Fig. 2C and Supporting Information Fig. 1B); the expression levels of NKG2A, KIR2DL4, and LILRB1 in NK cells are shown in Fig. 2C. After cocultured with allogeneic primary NK cells, DKO Jurkat CAR19 cells constitutively expressing the mBE and mBG fusion proteins were resistant to lysis by allogeneic primary NK cells (Fig. 2D and Supporting Information Fig. 2A).

### Generation, characteristics, and specific cytotoxicity of UCAR T‐19 cells

**Figure 3:**
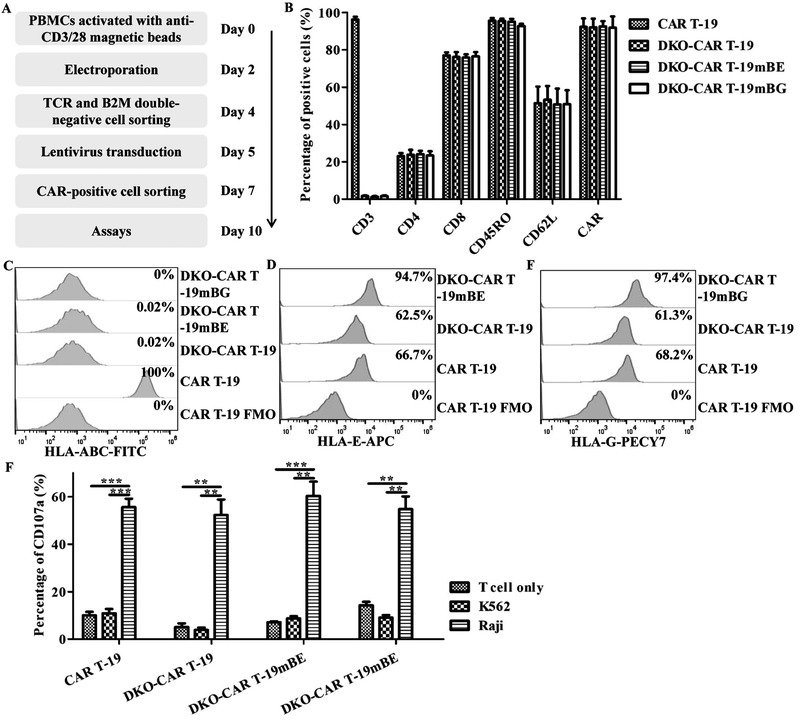
Characteristics of DKO gene‐edited CAR T‐19 cells. (A) DKO gene‐edited CAR T‐19 cells were produced and cultured for 10 days before being used in subsequent assays. (B) Phenotypes of DKO gene‐edited CAR T‐19 cells (*n* = 3 different donors in three independent experiments). (C) Representative HLA‐ABC expression in DKO gene‐edited CAR T‐19 cells. Gates were established by using FMO controls (HLA‐ABC). (D) Representative HLA‐E expression in DKO gene‐edited CAR T‐19 cells. Gates were established by using FMO controls (HLA‐E). (E) Representative HLA‐G expression in DKO gene‐edited CAR T‐19 cells. Gates were established by using FMO controls (HLA‐G). (F) CD107a expression in DKO gene‐edited CAR T‐19 cells after cocultured with CD19^+^ Raji cells (human Burkitt's lymphoma cell line) and CD19^−^ K562 cells (human chronic myelogenous leukemia cell line) at an effector‐to‐target ratio of 1:1 for 4 h (*n* = 3 different donors in three independent experiments. **P < 0.01, ***P < 0.001, *t*‐test, GraphPad Prism version 5).

To further confirm that constitutive expression of the mBE and mBG fusion proteins in UCAR T‐19 cells could reduce the risk of allogeneic NK‐cell mediated lysis, UCAR T‐19 cells were successfully generated from human peripheral blood mononuclear cells (PBMCs) according to standard operating procedures and analyzed after 10 days of culture (Fig. 3A). Negligible expression of CD3 and B2M was observed after electroporation with TRAC and B2M gRNA and the Cas9 protein and CD3 and B2M double‐negative cell sorting (Supporting Information Fig. 3A). CD3 expression was still nearly undetectable in DKO‐CAR T‐19, DKO‐CAR T‐19mBE, and DKO‐CAR T‐19mBG cells (Fig. 3B). No significant differences in the expression of other surface markers, including CD4, CD8, CD45RO, CD62L, and CAR, were observed among CAR T‐19, DKO‐CAR T‐19, DKO‐CAR T‐19mBE, and DKO‐CAR T‐19mBG cells (Fig. 3B and Supporting Information Fig. 3B). Like endogenous B2M, HLA‐ABC expression was nearly undetectable in DKO‐CAR T‐19, DKO‐CAR T‐19mBE, and DKO‐CAR T‐19mBG cells (Fig. 3C); however, HLA‐E and HLA‐G expression was observed in DKO‐CAR T‐19 cells (Fig. 3D, and E). Importantly, HLA‐E and HLA‐G exhibited higher percentages and MFI in DKO‐CAR T‐19mBE and DKO‐CAR T‐19mBG cells (Fig. 3D and E). CD107a expression was observed in CAR T‐19, DKO‐CAR T‐19, DKO‐CAR T‐19mBE, and DKO‐CAR T‐19mBG cells after coincubation with the CD19^+^ cell line Raji (Fig. 3F and Supporting Information Fig. 4), indicating that these cells had specific cytolytic activity against CD19‐positive tumor cells.

### Constitutive expression of the mBE and mBG fusion proteins in UCAR T‐19 cells prevented allogeneic NK cell‐mediated lysis and recognition by allogeneic T cells

**Figure 4:**
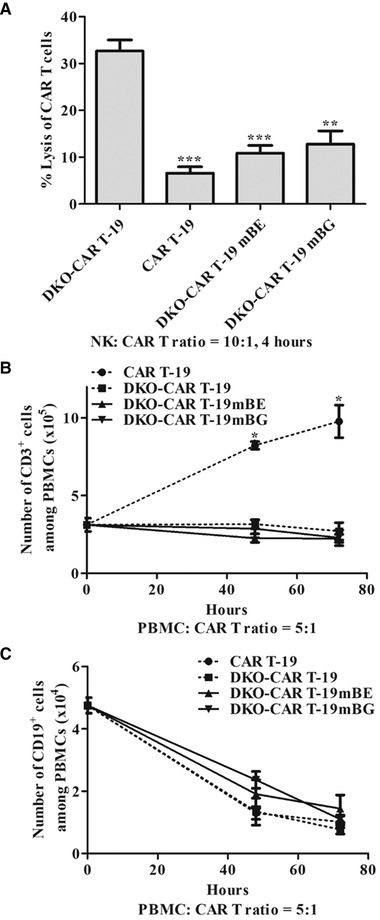
Constitutive expression of the mBE and mBG fusion proteins prevented allogeneic NK cell‐mediated lysis and recognition by allogeneic T cells. (A) Allogeneic NK cell cytotoxicity on DKO gene‐edited CAR T‐19 cells after coculture at an NK‐to‐CAR T cell ratio of 10:1 for 4 h (*n* = 4 different donors in four independent experiments. **P < 0.01, ***P < 0.001, versus DKO‐CAR T‐19, *t*‐test, GraphPad Prism version 5). (B) Recognition by allogeneic T cells. Allogeneic PBMCs cocultured with mitomycin C‐pretreated CAR T‐19 and DKO gene‐edited CAR T‐19 cells at a PBMC‐to‐CAR T‐19 cell ratio of 5:1 and the number of CD3^+^ cells were calculated for 48 and 72 h (*n* = 3 different donors in three independent experiments. *P < 0.05, CAR T‐19 versus DKO‐CAR T‐19, DKO‐CAR T‐19mBE or DKO‐CAR T‐19mBG, *t*‐test, GraphPad Prism version 5). (C) Specificity of DKO gene‐edited CAR T‐19 cells. Allogeneic PBMCs cocultured with CAR T‐19 and DKO gene‐edited CAR T‐19 cells at a PBMC‐to‐CAR T‐19 cell ratio of 5:1 and the number of CD19^+^ cells were calculated for 48 and 72 h (*n* = 3 different donors in three independent experiments).

To further clarify whether the constitutive expression of the mBE and mBG fusion proteins in UCAR T‐19 cells could prevent allogeneic NK cell‐mediated lysis, UCAR T‐19 cells were cocultured with allogeneic NK cells at an NK to UCAR T‐19 ratio of 10:1. After coculture for 4 and 24 h, significantly more DKO‐CAR T‐19 cells were lyzed by allogeneic NK cells than CAR T‐19 cells (Fig. 4A and Supporting Information Fig. 2B). In addition, the lysis by allogeneic NK cells was significantly decreased in DKO‐CAR T‐19 cells constitutively expressing the mBE and mBG fusion proteins compared with DKO‐CAR T‐19 cells (Fig. 4A and Supporting Information Fig. 2B).

In addition, we used an in vitro model to assess whether allogeneic T cells could recognize and respond to UCAR T‐19 cells expressing the mBE and mBG fusion proteins. Normal allogeneic human PBMCs (Supporting Information Fig. 5) were cocultured with UCAR T‐19 cells pre‐exposed to mitomycin C. The number of CD3^+^ T cells among PBMCs was significantly increased after cocultured with CAR T‐19 cells compared with DKO‐CAR T‐19 cells and DKO‐CAR T‐19 cells constitutively expressing the mBE and mBG fusion proteins (Fig. 4B), indicating that the DKO‐CAR T‐19 cells and DKO‐CAR T‐19 cells expressing the mBE and mBG fusion proteins could not activation‐induce the proliferation of allogeneic T cells and that constitutive expression of the mBE and mBG fusion proteins prevented the recognition by allogeneic T cells. In addition, the number of CD19^+^ cells among PBMCs was decreased after cocultured with UCAR T‐19 cells, indicating the specific lysis by UCAR‐19 cells that expressing the mBE and mBG fusion proteins (Fig. 4C).

## Discussion

Elimination of mismatched HLA and TCR expression in donor‐derived transplanted cells without the need to suppress host immunity or limit transplanted cells migrating to privileged sites to avoid immune surveillance could prevent the allogeneic response of transplanted cells [10‐12, 16]. Therefore, we herein successfully manufactured UCAR T‐19 cells using gene‐editing technologies to reduce the allogeneic response and revealed the constitutive expression of the mBE and mBG fusion proteins in UCAR T‐19 cells could effectively prevent allogeneic NK cell‐mediated lysis.

Gene‐editing technologies play a crucial role in UCAR T cell manufacture, and the effective editing of multiple genes in human primary T cells is not possible with the currently available methods due to a low gene‐editing efficacy or high transfection‐associated toxicity [21]. Recent studies have indicated that the CRISPR/Cas9 system is a potential alternative gene‐editing technology for targeted gene engineering and is powerful in human T cells with high gene‐knockout efficacy [10, 22, 23]. Lentiviral and adenoviral strategies have limited gene disruption abilities in T cells due to the low transfection efficacies of Cas9 and gRNA [24, 25]. Therefore, in this study, UCAR T cells were manufactured with TRAC and B2M gRNA and the Cas9 protein electroporation [26]. This protocol led to the effective disruption of TCR and HLA class I molecule expression in CAR T cells and Jurkat cells. In addition, our data indicated that the elimination of TCR and HLA class I molecules significantly reduces the allogeneic response of UCAR T cells. Additional rigorous assessments in animal models should be performed to confirm the safety of the method and to the allogeneic response of these genetically edited T cells.

Recent studies indicated that disruption of endogenous TCR expression in CAR T cells could significantly reduce the occurrence of GvHD [7, 8, 9], And several other studies have demonstrated that elimination of HLA class I molecule expression reduces the alloreactivity of T cells [10, 11, 12]. However, allogeneic NK cells can still kill HLA class I‐negative cells, including UCAR T cells, due to the absence of killer Ig‐like receptor (KIR) ligands [10, 13, 14]. Several studies have indicated that lymphodepletion via chemotherapy or NK cell‐specific antibody can effectively deplete most NK cells, thereby potentially reducing the NK cell‐mediated lysis of HLA class I‐negative cells [27, 28]. However, these regimens may be toxic and affect the anti‐tumor activity of UCAR T cells. Several recent studies have indicated that the constitutive expression of nonclassical HLA class I molecules such as HLA‐E and HLA‐G represents a potential solution for protecting HLA class I‐negative cells against allogeneic NK cell‐mediated lysis [15, 16, 29]. Therefore, in this study, the mBE and mBG fusion proteins were constitutively expressed in UCAR T‐19 cells via lentiviral delivery. Indeed, we validated that the NK cell‐mediated lysis of UCAR T‐19 cells constitutively expressing the mBE or mBG fusion protein was reduced, suggesting that this approach can be used to prevent NK cell‐mediated lysis after UCAR T cell treatment.

In this study, we disrupted the expression of HLA class I molecules by knocking out endogenous B2M. The expression of polymorphic HLA class I molecules, including HLA‐A, HLA‐B, and HLA‐C, was decreased in both Jurkat cells and human T cells, consistent with the expression of B2M; however, HLA‐E and HLA‐G were still detectable in endogenous B2M‐negative cells. Lysis induced by allogeneic NK cells was not prevented in Jurkat or CAR T‐19 cells lacking B2M, in which HLA‐E or HLA‐G was still detectable. B2M‐free heavy‐chain HLA‐G molecules are found in the placenta and cannot be recognized by LILRB1, while B2M‐free heavy‐chain HLA‐G molecules are expressed on the cell surface [30]. In addition, previous studies analyzed the physiological importance of MHC class I molecules protecting against NK cell‐mediated lysis and indicated that B2M‐free heavy‐chain MHC class I molecules could not sufficiently prohibit NK cell‐mediated lysis [31, 32]. Overall, B2M‐dependent MHC class I molecules are recognizable by NK cell inhibitory receptors, thereby protecting against NK cell‐mediated lysis.

In conclusion, our data indicate that CAR T cells lacking the TCR and B2M potent antitumor activity and reduced allogeneic responses can be successfully manufactured using CRISPR/Cas9 gene‐editing technology. In addition, these cells can reduce allogeneic NK cell‐mediated lysis by constitutively expressing the mBE and mBG fusion proteins. Therefore, UCAR T‐19 cells maybe an alternative to autologous CAR T cells and have unexpected outcomes in the clinic.

## Materials and methods

### Cell lines

The human acute T cell leukemia cell line Jurkat was purchased from ATCC and maintained in RPMI 1640 medium (Gibco) supplemented with 10% (v/v) FBS (Gibco), and 100 U/mL penicillin/streptomycin (Gibco).

### Manufacture of CAR T‐19 cells

CAR T‐19 cells were generated from healthy volunteer donor PBMCs according to standard operating procedures [33]. Primary T cells from PBMCs were activated using Dynabead Human T‐Activator CD3/CD28 magnetic beads (Invitrogen) and cultured in X‐VIVO 15 medium (Lonza) supplemented with 300 U/mL recombinant human IL‐2 (PeproTech). Lentiviral supernatants were loaded on RetroNectin (Takara)‐coated plates, and the plates were centrifuged at 2000 × *g* for 2 h at 32°C. Then, activated T cells were added to the plates and centrifuged at 800 × *g* for 10 min. The transduced cells were collected and transferred into new flasks for further expansion on the following day. The cells were cultured at 1 × 10^6^ cells per milliliter in X‐VIVO 15 medium supplemented with 300 IU/mL IL‐2 and harvested on day 10.

### Electroporation

The Cas9 protein and sgRNA were electroporated into Jurkat cells and human primary T cells to knock out the expression of endogenous TCR and B2M as previously described [21, 26]. To prepare the ribonucleoprotein mixture, 6 μg of the Cas9 protein (Integrated Device Technology, Inc.) and 6 μg of sgRNA (Integrated Device Technology, Inc.) were incubated together at room temperature for 20 minutes immediately before electroporation. Jurkat and T cells (5 × 10^5^) were centrifuged and resuspended in 50 μL of Opti‐MEM medium (Gibco) containing the ribonucleoprotein mixture and transferred into a 2 mm cuvette (Harvard Apparatus BTX). Then, the cells were electroporated using a BTX Gemini System (Harvard Apparatus BTX) at 250 V for 5 ms. Following electroporation, the cells were immediately transferred into 2 mL of prewarmed medium and cultured at 37°C and 5% CO_2_. The gRNA targeting sequences used in this study were as follows: TRAC gRNA: AGAGTCTCTCAGCTGGTACA and B2M gRNA: CGCGAGCACAGCTAAGGCCA [10].

### CAR‐positive cell sorting

Cells were incubated with a biotin‐SP‐AffiniPure F(ab)’2 fragment‐specific goat anti‐mouse IgG antibody (Jackson ImmunoResearch) and then with streptavidin‐phycoerythrin (PE) (BD Biosciences). Then, the cells were incubated with anti‐PE microbeads at 4°C for 15 min, and magnetic separation was performed to collect CAR‐positive cells according to the manufacturer's protocol (Miltenyi Biotec).

### TCR and B2M double‐negative cell sorting

After electroporation, cells double‐negative for TCR and B2M were sorted according to the manufacturer's protocol (Miltenyi Biotec). First, the cells were incubated with anti‐human PE‐conjugated B2M antibody (BD Biosciences) at 4°C for 10 min, followed by incubation with anti‐PE microbeads at 4°C for 15 min and magnetic separation was performed to collect B2M‐negative cells according to the manufacturer's protocol. Then, B2M‐negative cells were sequentially incubated with an anti‐human FITC‐conjugated CD3 antibody (BD Biosciences) at 4°C for 10 min and with anti‐FITC microbeads at 4°C for 15 min, and CD3‐negative cells were collected after magnetic separation.

### Isolation of primary NK cells

Primary NK cells were isolated from healthy volunteer donor PBMCs according to the manufacturer's protocol (Miltenyi Biotec). PBMCs were sequentially incubated with an anti‐human PE‐conjugated CD56 antibody (BD Biosciences) at 4°C for 10 minutes and anti‐PE microbeads at 4°C for 15 min, and magnetic separation was then performed according to the manufacturer's protocol.

### Flow cytometry

Gene‐edited Jurkat CAR19 and CAR T‐19 cells were incubated with a biotin‐SP‐AffiniPure F(ab)’2 fragment‐specific goat anti‐mouse IgG antibody (Jackson ImmunoResearch) to assess CAR expression, followed by incubation with streptavidin‐PE (BD Biosciences). In addition, the gene‐edited Jurkat CAR19 and CAR T‐19 cells were incubated with anti‐human B2M, CD3 (BD Biosciences), HLA‐ABC, HLA‐C, HLA‐E, and HLA‐G (Biolegend) antibodies.

Gene‐edited CAR T‐19 cells were washed and stained with anti‐human CD3, CD4, CD8, CD45RO, and CD62L antibodies (BD Biosciences).

Gene‐edited CAR T‐19 cells were cocultured with Raji or K562 cells at an effector‐to‐target (E:T) ratio of 1:1 together with an anti‐human CD107a antibody (BD Biosciences) for 1 h to assess degranulation, followed by incubation with a Golgi Plug protein transport inhibitor (BD Biosciences) for 3 h.

Primary NK cells were incubated with anti‐human CD3, CD56 (BD Biosciences), NKG2A, KIR2DL4, and LILRB1 (Biolegend) antibodies.

All of the antibody information is presented in Supporting Information Table 1. All samples were assessed using DxFLEX flow cytometry (Beckman Coulter). The data were analyzed with Kaluza Analysis 2.0 (Beckman Coulter) and FlowJo software version 10 (FlowJo LLC).

### In vitro cytotoxicity of NK cells

To assess the ability of the mBE and mBG fusion proteins to protect against allogeneic NK cell‐mediated lysis, gene‐edited Jurkat CAR19 cells were cocultured with primary NK cells at effector‐to‐target ratios of 5:1, 2.5:1, 1.25:1, 0.625:1, and 0.3125:1 for 4 and 24 h. Gene‐edited CAR T‐19 cells were cocultured with allogeneic primary NK cells at an effector‐to‐target ratio of 10:1 for 4 and 24 h. Primary NK cells were used directly after CD56‐positive cell isolation, and cytotoxicity was evaluated with an apoptosis detection kit (BD) using DxFLEX flow cytometry.

### Detection for recognition by allogeneic T cells

Allogeneic PBMCs were first labeled with 5 μM carboxyfluorescein succinimidyl ester (CFSE) (Dojindo Laboratories) at room temperature for 10 min and then cocultured with CAR T‐19 and DKO gene‐edited CAR T‐19 cells pre‐exposed to 10 μg/mL mitomycin C at a PBMC‐to‐CAR T‐19 cell ratio of 5:1. After cocultured for 48 and 72 h, the percentage of CFSE^+^CD3^+^ cells among the PBMCs was determined by flow cytometry, and the total number of viable cells was determined by 0.4% trypan blue staining. The number of CD3^+^ cells among the PBMCs was determined by the following equation: CD3^+^ cell number = percentage of CFSE^+^CD3^+^ cells × total number of viable cells. In addition, the number of CD19^+^ cells among the PBMCs was determined described above to analyze the specificity of gene‐edited CAR T‐19 cells.

### Statistical analysis

Statistical analyzes were performed with GraphPad Prism version 5 for Windows. The results are shown as the means ± SDs. The significance of differences between groups was determined by *t*‐tests, and *P*‐values < 0.05 were considered statistically significant.

## Author Contributions

W.H., Y.W., H.W., and Y.G. conceived and designed the experiment; Y.G., B.X., Z.W., C.T., and D.C. performed the experiments; Y.G. and B.X. analyzed the data; J.W. processed and typeset figures; Y.G. wrote the manuscript, and Y.G., J.B. and W.H. revised the manuscript. All authors read and approved the final manuscript.

## Conflict of Interest

The authors declare no commercial or financial conflict of interest.

### Peer review

The peer review history for this article is available at https://publons.com/publon/10.1002/eji.202049107.

**Abbreviations**
B2M: beta‐2 microglobulin
CAR T: chimeric antigen receptor‐modified T
CAR: chimeric antigen receptor
DKO: double‐knockout
FMO: fluorescence‐minus‐one
GvHD: graft versus host disease
HLA: human leukocyte antigen
KIR: killer Ig‐like receptor
mBE: mutant B2M‐HLA‐E
mBG: mutant B2M‐HLA‐G
MFI: mean fluorescence intensity
NK: natural killer
PBMCs: peripheral blood mononuclear cells
TCRs: T cell receptors
UCAR T: universal chimeric antigen receptor‐modified T

## Supporting information

Supporting information

## Data Availability

The data that support the findings of this study are available from the corresponding author upon reasonable request.
