# Supplementary material for: Mutant B2M‐HLA‐E and B2M‐HLA‐G fusion proteins protects universal chimeric antigen receptor‐modified T cells from allogeneic NK cell‐mediated lysis
Source: Eur J Immunol. 2021 Aug 19;51(10):2513–21. doi: 10.1002/eji.202049107 (PMC9292285; doi:10.1002/eji.202049107)
Supplement: Supplementary file 1 — Supporting information [file EJI-51-2513-s001.pdf]

Mutant B2M-HLA-E and B2M-HLA-G fusion proteins protects universal chimeric antigen receptor-modified T cells from allogeneic NK cell-mediated lysis

Supplemental Figures:

Supplemental Figure 1

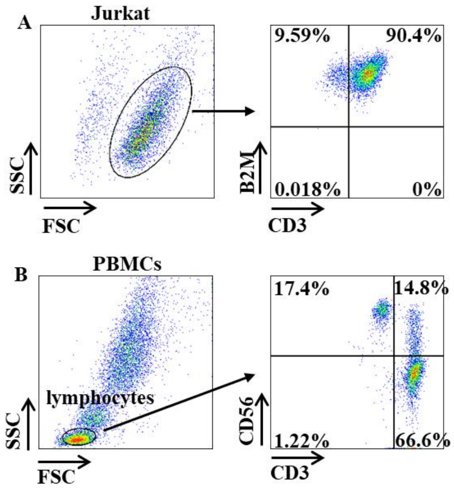

Supplemental Figure 1 A Representative gating strategy of TCR and B2M expression in Jurkat cells. B Representative gating strategy of CD3 and CD56 expression in PBMCs. The numbers represent the percentages of positive cells. SSC, side scatter; FSC, forward scatter.

Supplemental Figure 2

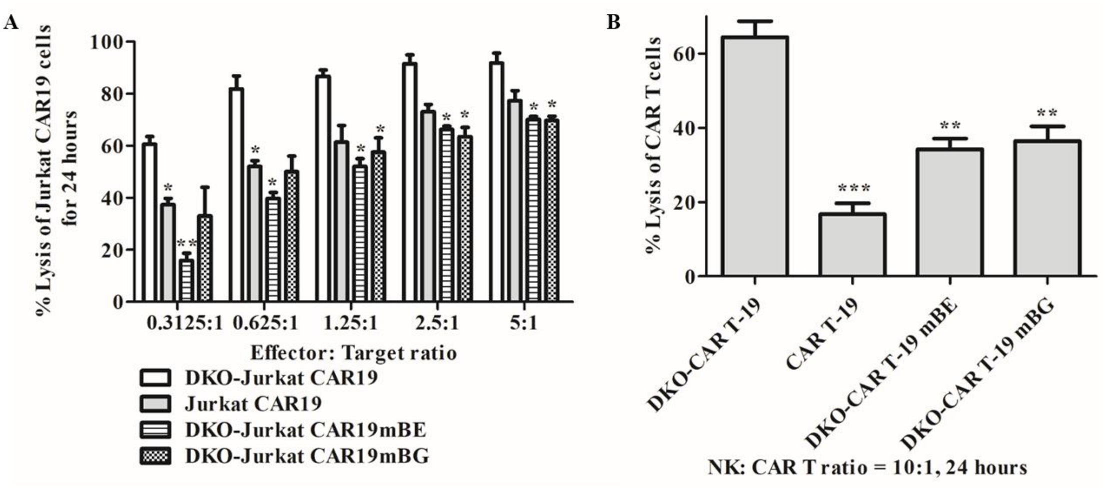

Supplemental Figure 2 **A** NK cell cytotoxicity on DKO gene-edited Jurkat CAR19 cells after cocultured at effector-to-target ratios of 5:1, 2.5:1, 1.25:1, 0.625:1 and 0.3125:1 for 24 hours (n = 3 different donors in three independent experiments. *, P<0.05, **, P<0.01, versus DKO-Jurkat CAR19; t test, GraphPad Prism version 5). **B** Allogeneic primary NK cell cytotoxicity on DKO gene-edited CAR T-19 cells after cocultured at a NK-to-CAR T cell ratio of 10:1 for 24 hours (n = 4 different donors in four independent experiments. **, P<0.01, ***, P<0.001, versus DKO-CAR T-19; t test, GraphPad Prism version 5).

Supplemental Figure 3

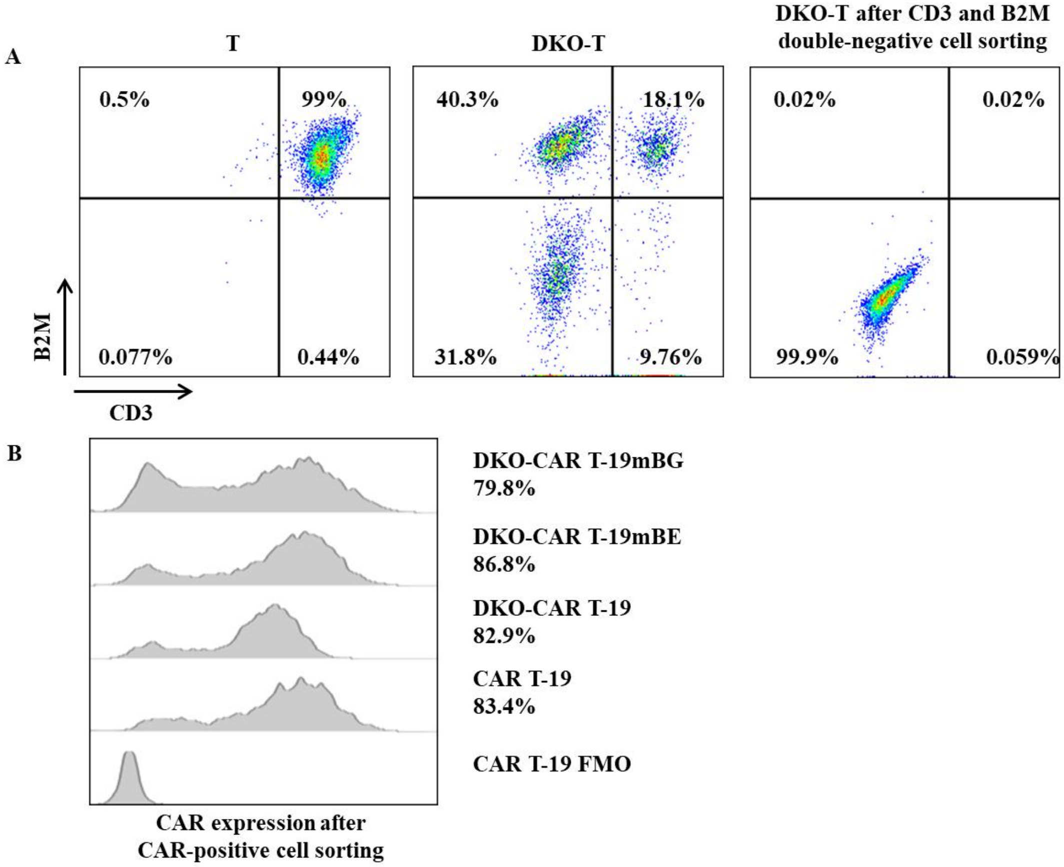

Supplemental Figure 3 Generation of TCR and B2M double-negative knockout (DKO) gene-edited CAR T-19 cells. **A** Representative TCR and B2M expression in T cells before and after electroporation and in DKO-T cells after TCR and B2M double-negative cell sorting. The numbers represent the percentages in each gate. **B** Representative CAR expression in gene-edited CAR T-19 cells after CAR-positive cell sorting. Gates were established by using FMO controls (biotin-SP-AffiniPure F(ab)'2 fragment). The numbers represent the percentages of positive cells.

Supplemental Figure 4

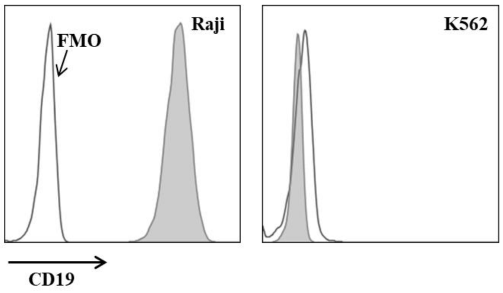

Supplemental Figure 4 CD19 expression in Raji (human Burkitt's lymphoma cell line) and K562 (human chronic myelogenous leukemia cell line) cells as determined by flow cytometry. These tumor cells were incubated with an anti-human CD19 antibody (BD), and gates were established by using FMO controls (CD19).

Supplemental Figure 5

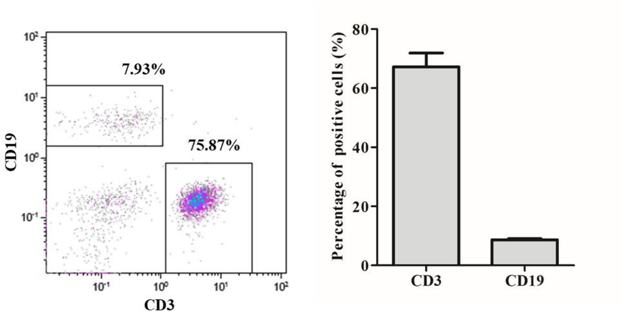

Supplemental Figure 5 CD3 and CD19 expression in PBMCs obtained from volunteer donors. Representative CD3 and CD19 expression in PBMCs is shown in the left panel. The numbers represent the percentages of positive cells. The statistical analysis of CD3 and CD19 expression in PBMCs is shown on the right. The bars in the right panel show the mean ± SDs from 3 donors in three independent experiments.

Supplemental Table 1 Information of flow antibodies

Company	Antigen	Fluorescence	Category number
BD	CD3	FITC	555332
	CD3	PerCP	347344
	CD3/4/8	PerCP/FITC/PE	79978
	CD19	PE	340364
	CD45RO	Alexa Fluor ® 700	561136
	CD56	PE	561903
	CD62L	APC	559772
	CD107a	APC	560664
	B2M	PE	551337
	streptavidin	PE	554061
Jackson ImmunoResearch	biotin-SP-AffiniPure F(ab)'2 fragment-specific goat anti-mouse IgG antibody	Not application	115065072
Biolegend	HLA-ABC	FITC	311404
	HLA-C	Not application	373302
	HLA-E	APC	342606
	HLA-G	PECY7	335912
	NKG2A	APC	375107
	KIR2DL4	APC	247007
	LILRB1	APC	333719
